# Physician Preferences in Using Novel Digital Devices for the Management of Atrial Fibrillation—A DAS‐CAM III Survey

**DOI:** 10.1002/clc.24331

**Published:** 2024-11-25

**Authors:** Martin Manninger, David Zweiker, Tatevik Hovakimyan, Paweł T. Matusik, Sergio Conti, Pierre Ollitrault, Aapo Aro, Bart A. Mulder, Wolfgang Dichtl, Christian‐Hendrik Heeger, Rachel ter Bekke, Enes Elvin Gul, Bob Weijs, Ann‐Kathrin Rahm, Angeliki Darma, Banu Evranos, Avi Sabbag, Kgomotso Moroka, Vassil Traykov, Jacob Moesgaard Larsen, Gisella Rita Amoroso, Stijn Evens, William F. McIntyre, Dominik Linz

**Affiliations:** ^1^ Department of Internal Medicine, Division of Cardiology Medical University of Graz Graz Austria; ^2^ Department of Cardiology, Cardiovascular Research Institute Maastricht (CARIM) Maastricht University Medical Centre Maastricht The Netherlands; ^3^ Department of Cardiology and Intensive Care Clinic Ottakring Vienna Austria; ^4^ Department of Cardiac Arrhythmology Nork‐Marash Medical Center Yerevan Armenia; ^5^ Department of Electrocardiology, Institute of Cardiology Jagiellonian University Medical College, Faculty of Medicine Kraków Poland; ^6^ Department of Electrocardiology The John Paul II Hospital Kraków Poland; ^7^ Department of Cardiac Electrophysiology ARNAS Civico Hospital Palermo Italy; ^8^ Electrophysiology Unit, Department of Cardiology Caen University Hospital Caen France; ^9^ Division of Cardiology, Heart and Lung Center University of Helsinki and Helsinki University Hospital Helsinki Finland; ^10^ Department of Cardiology, University Medical Centre Groningen University of Groningen Groningen The Netherlands; ^11^ Department of Internal Medicine III Medical University of Innsbruck Innsbruck Austria; ^12^ Department of Rhythmology, University Heart Center Lübeck University Hospital Schleswig‐Holstein Lübeck Germany; ^13^ German Center for Cardiovascular Research (DZHK), Partner Site Lübeck Germany; ^14^ Division of Cardiac Electrophysiology Madinah Cardiac Center Madinah Saudi Arabia; ^15^ Medicine Hospital Istanbul Atlas University Istanbul Turkey; ^16^ Clinic for Internal Medicine, Cardiology and Electrophysiology Katholische Stiftung Marienhospital Aachen Germany; ^17^ Department of Cardiology, Angiology and Pneumology Heidelberg University Hospital Heidelberg Germany; ^18^ Heidelberg Center for Heart Rhythm Disorders (HCR) Heidelberg University Hospital Heidelberg Germany; ^19^ German Center for Cardiovascular Research (DZHK) Partner Site Heidelberg/Mannheim Heidelberg Germany; ^20^ Department of Electrophysiology Heart Centre Leipzig Leipzig Germany; ^21^ Department of Cardiology Faculty of Medicine Hacettepe University Ankara Turkey; ^22^ Leviev Heart Center, Sheba Medical Center, Affiliated With Sackler School of Medicine Tel Aviv University Tel Aviv Israel; ^23^ Department of Cardiology, Universitas Academic Hospital University of the Free State Bloemfontein South Africa; ^24^ Department of Invasive Electrophysiology and Cardiac Pacing Acibadem City Clinic Tokuda Hospital Sofia Bulgaria; ^25^ Department of Cardiology Aalborg University Hospital Aalborg Denmark; ^26^ Department of Clinical Medicine Aalborg University Aalborg Denmark; ^27^ Dipartimento Medico Specialistico, Divisione di Cardiologia Ospedale “SS Annunziata” Savigliano Italy; ^28^ Qompium Hasselt Belgium; ^29^ Population Health Research Institute Hamilton Canada; ^30^ Department of Medicine McMaster University Hamilton Canada; ^31^ Department of Biomedical Sciences, Faculty of Health and Medical Sciences University of Copenhagen Copenhagen Denmark

**Keywords:** atrial fibrillation, digital devices, photoplethysmography, rhythm monitoring, screening, wearables

## Abstract

**Aim:**

A recent European Heart Rhythm Association (EHRA) practical guide provides guidance on the use of novel digital devices for heart rhythm analysis using either electrocardiogram (ECG) or photoplethysmography (PPG) technology for the diagnosis of atrial fibrillation (AF). This survey assesses physicians' preferences to use digital devices in patients with possible AF and their impact on clinical decision‐making.

**Methods and Results:**

Participants of the DAS‐CAM III initiated and distributed an online survey assessing physician preferences in using digital devices for the management of AF in different clinical scenarios. A total of 505 physicians (median age: 38 [IQR 33–46] years) from 30 countries completed the survey. A third of respondents were electrophysiologists, the others were cardiologists, cardiology residents, or general practitioners. Electrophysiologists were more likely to have experience with both ECG‐based (92% vs. 68%, *p* < 0.001) and PPG‐based (60% vs. 34%, *p* < 0.001) digital devices. The initial diagnostic approach to each scenario (symptomatic low‐risk, symptomatic high‐risk, or asymptomatic high‐risk patient) was heterogeneous. Electrophysiologists preferred intermittent single‐lead ECG monitoring to traditional Holter ECGs to screen for AF. Both electrophysiologists and non‐electrophysiologists would rarely use PPG‐based devices to diagnose and screen for AF (8.2%–9.8%). Electrophysiologists and non‐electrophysiologists use ECG‐based technology to confirm PPG‐documented tracings suggestive of AF.

**Conclusion:**

While PPG‐based digital devices are rarely used for diagnosis and screening for AF, intermittent ECG‐based digital devices are beginning to be implemented in clinical practice. More education on the potential of novel digital devices is required to achieve diagnostic pathways as suggested by the EHRA practical guide.

## Graphical abstract

This survey of > 500 cardiologists and electrophysiologists outlines physician's preferences in using novel digital rhythm devices in the management of atrial fibrillation. The survey consisted of 22 case‐based questions. Currently, physicians rarely use photoplethysmography‐based devices. However, the uptake of ECG‐based devices is high, especially in electrophysiologists.

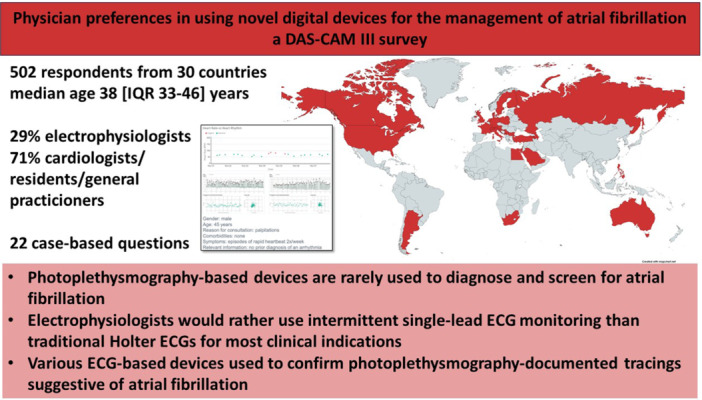

## 1 Introduction

Technological advances in digital devices using either electrocardiogram (ECG) or photoplethysmography (PPG) signals to assess heart rate and rhythm have led to a rapid uptake of these devices in clinical practice for arrhythmia diagnosis and remote management of patients with arrhythmias [1, 2, 3, 4]. European Society of Cardiology (ESC) guidelines continue to require ECG documentation for the diagnosis of rhythm disorders, including atrial fibrillation (AF). Therefore, devices using ECG technology remain the gold standard for arrhythmia detection. However, PPG‐based consumer‐facing technology (e.g., smartphones, smartwatches) offers the advantages of wide availability, ease of use, and ability to perform continuous monitoring (e.g., by a smartwatch) [5, 6, 7]. Although PPG‐based digital devices and accompanying algorithms have been validated for AF detection and heart rate assessment during sinus rhythm and AF, the uptake of PPG‐based devices into clinical practice remains limited [4, 8, 9, 10]. In 2020, the wEHRAbles surveys demonstrated that clinicians are aware of novel digital devices and that they are used in routine clinical practice, but ECG‐based devices are used almost exclusively in preference to PPG‐based devices [3, 4]. In 2022, the European Heart Rhythm Association (EHRA) published a practical guide for the use of digital devices for arrhythmia management [1]. This guide stated that both PPG‐ and ECG‐based digital devices can be used for arrhythmia detection in symptomatic patients in both AF screening and AF management. Despite increasing evidence for PPG‐based devices, current physician preferences in using novel digital devices for the management of AF are unknown.

The Diploma of Advanced Studies in Cardiac Arrhythmia Management III (DAS‐CAM III) participants initiated and conducted a survey to assess how physicians use digital devices in patients with possible AF and to identify the impact of data derived from ECG‐ and PPG‐based digital devices on clinical decision‐making.

## 2 Materials and Methods

An online questionnaire consisting of 22 questions was distributed by EHRA DAS‐CAM III participants via their networks as well as via social media platforms (Twitter, LinkedIn, and Facebook). The questionnaire included questions on demographics (age, location, and current profession), experience with ECG‐ and PPG‐based rhythm monitoring devices, and three specific patient scenarios. The scenarios, described in Table 1, included (1) a symptomatic low‐risk patient (a 45‐year‐old male with CHA_2_DS_2_‐VASc = 0 with a rapid heartbeat twice per week), (2) an asymptomatic high‐risk patient (a 70‐year‐old female with hypertension and type II diabetes mellitus [CHA_2_DS_2_‐VASc = 4]), and (3) a symptomatic high‐risk patient (a 70‐year‐old female patient with hypertension and type II diabetes mellitus [CHA_2_DS_2_‐VASc 4] with episodes of rapid heartbeat twice per week). Participants could choose from different monitoring strategies as follows: 12‐lead ECG, Holter ECG for 24 h, Holter ECG for > 24 h, and on‐demand intermittent monitoring with either single‐lead ECG‐based or PPG‐based device or no monitoring and clinical follow‐up. After selecting a monitoring strategy, participants were asked to select a second test from the same list after the first test failed to document an arrhythmia. For each scenario, when selecting PPG workup, three different results were generated using the FibriCheck database (Qompium, Hasselt, Belgium): uneventful measurements, tracings consistent with paroxysmal AF, and tracings consistent with persistent AF (see Supporting Information S1: Table 1). All questions, besides questions on demographics, were classified as mandatory.

**Table 1:** Case scenarios.

	Case 1: Symptomatic low‐risk patient	Case 2: Asymptomatic high‐risk patient	Case 3: Symptomatic high‐risk patient
Gender	Male	Female	Female
Age (years)	45	70	70
Reason for consultation	Palpitations	Regular follow‐up	Palpitations
Comorbidities	None	Hypertension, type II diabetes mellitus	Hypertension, type II diabetes mellitus
Symptoms	Rapid heartbeat 2×/week	None	Rapid heartbeat 2×/week
CHA_2_DS_2_‐VASc	0	4	4

Continuous variables are presented as mean ± SD or median (interquartile range [IQR]). Categorical variables are presented as percentages and counts. Questions on clinical decision‐making were compared using Wilcoxon's test for matched samples and Mann–Whitney *U*‐test for independent variables. Categorical variables were compared using the chi‐squared test. A two‐sided *p* value of < 0.05 was considered significant. Sankey plots were used to visualize respondent's device selections at two time points. Statistical analyses were performed using R version 4.3.1 (The R Foundation, Vienna, Austria).

## 3 Results

**Figure 1:**
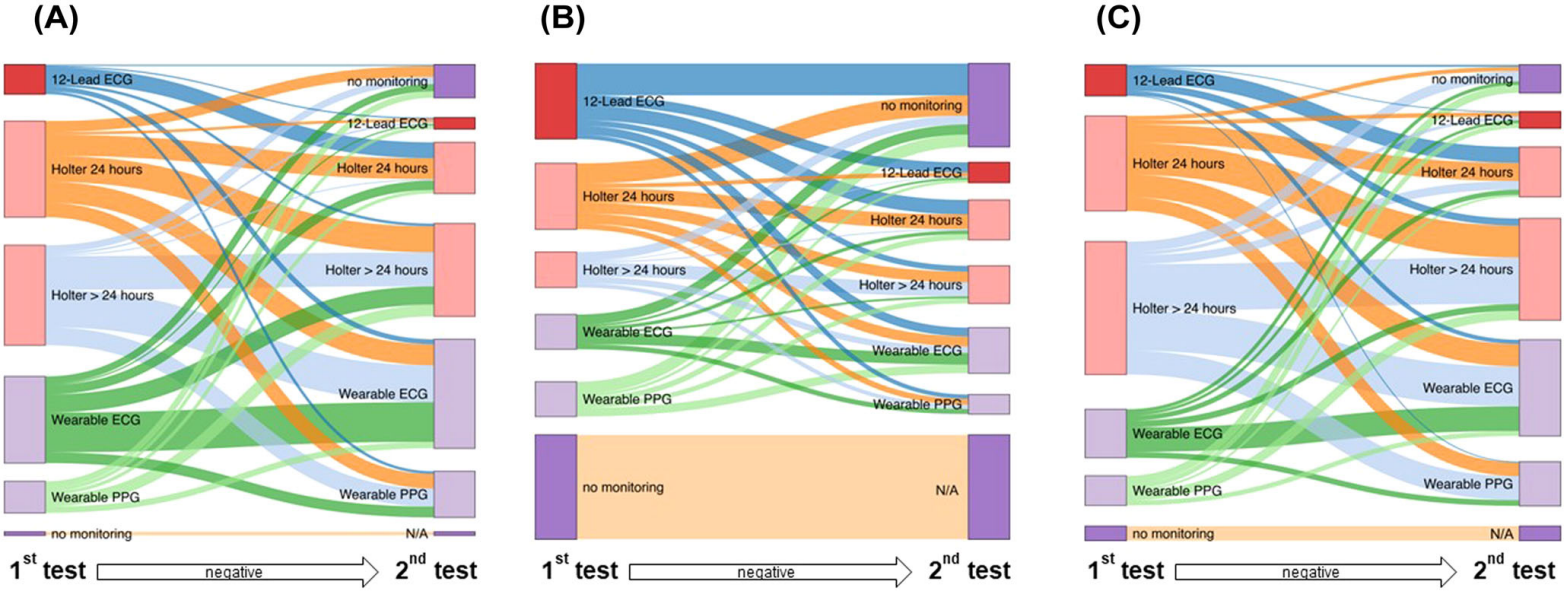
Sankey plots showing first (left side) and second choices (if first test was negative, right side) of diagnostic tools for the three case scenarios of a symptomatic low‐risk (A), asymptomatic high‐risk (B), and symptomatic high‐risk patient (C). If participants chose no monitoring, they did not proceed with the second question (N/A).

A total of 563 respondents (median age 38 [IQR 33–46] years) from 30 countries participated in the survey. From these, 61 were excluded because of missing data (*n* = 6) and/or professions other than physicians (*n* = 58) (see Supporting Information S1: Figure 1). A third of respondents (*n* = 146/502, 29%) were electrophysiologists and 71% were cardiologists, cardiology residents, or general practitioners. Electrophysiologists were more likely to have experience with ECG‐based (92% vs. 68%, *p* < 0.001) or PPG‐based (60% vs. 34%, *p* < 0.001) digital devices than non‐electrophysiologists. Experience with any wearable technology (i.e., PPG or ECG) was significantly associated with higher age (39 [34–46] vs. 37 [31–46] years, *p* = 0.042). A total of 76.5% of respondents were from Europe, 20.9% from Asia, 1.4% from the Americas, 1.0% from Africa, and 0.2% from Australia. Respondents from Asia had significantly less experience in wearable technology (51.4%) than respondents from Europe (83.6%) or other continents (76.9%, *p* < 0.001).

### 3.1 Surveyed Case Scenarios

#### 3.1.1 Case 1: Symptomatic Low‐Risk Patient

When presented with a case of a 45‐year‐old male patient without comorbidities (CHA_2_DS_2_‐VASc = 0) reporting episodes of rapid heartbeat twice per week, most respondents would select ECG‐based monitoring (28.8% Holter for > 24 h, 27.8% Holter for 24 h, and 25.2% single‐lead ECG‐based digital device). Only 8.9% of participants would start on‐demand intermittent monitoring by a PPG‐based digital device (see Figure 1A). Physicians experienced with PPG are more likely to use a PPG‐based device in this scenario, compared to physicians without PPG experience (12.1% vs. 6.6%, *p* = 0.026). Electrophysiologists were more likely to select intermittent monitoring via a single‐lead ECG‐based digital device (48.1% vs. 26.3%, *p* < 0.001) than non‐electrophysiologists.

**Figure 2:**
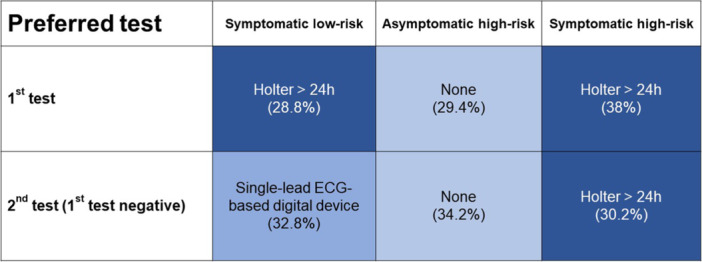
Preferred first and second diagnostic tests for each case scenario.

If a first test failed to document an arrhythmia, one‐third of respondents (32.8%) would select intermittent monitoring via a single‐lead ECG‐based digital device, 25.9% of respondents would select a Holter for > 24 h, 15.2% of respondents would select a Holter for 24 h, and 14.8% of respondents would select monitoring by a PPG‐based digital device (see Figure 2).

#### 3.1.2 Case 2: Asymptomatic High‐Risk Patient

When presented with a case of a 70‐year‐old female patient with hypertension and type II diabetes mellitus (CHA_2_DS_2_‐VASc = 4), who has no arrhythmia‐related symptoms, the highest proportion of respondents would perform no monitoring and follow‐up after 1 year (29.4%), while 21.7% would schedule a follow‐up consultation with an a 12‐lead resting ECG examination (see Figure 1B). Nineteen percent would perform 24 h Holter, 10.2% would use single‐lead ECG monitors, and 10.2% would perform a Holter for > 24 h. Only 9.8% of respondents would start on‐demand intermittent monitoring by a PPG‐based digital device. Physicians experienced with PPG would rather use a PPG‐based device in this scenario (13.6% vs. 7.2%, *p* = 0.026).

If a first test failed to document an arrhythmia, one‐third (34.2%) of respondents would not perform any other monitoring and schedule a 1‐year follow‐up consult, 17.4% of respondents would start intermittent monitoring by a single‐lead ECG‐based digital device, 16.1% of respondents would select 24 h Holter monitoring, 15.1% of respondents would select Holter monitoring for > 24 h, and 9.1% of respondents would select monitoring by a PPG‐based digital device (see Figure 2).

#### 3.1.3 Case 3: Symptomatic High‐Risk Patient

When presented with a case of a 70‐year‐old female patient with hypertension and type II diabetes mellitus (CHA_2_DS_2_‐VASc 4), who reports episodes of rapid heartbeat twice per week, 38% of respondents would schedule > 24 h Holter examination (see Figure 1C), 27% of respondents would perform Holter for 24 h, 14.1% respondents would use monitoring by a single‐lead ECG‐based digital device, and 8.7% of respondents would schedule a follow‐up consultation for an additional resting 12‐lead ECG examination. Only 8.2% of respondents would start on‐demand intermittent PPG monitoring.

If a first test failed to document an arrhythmia, most respondents (30.2%) would select > 24 h Holter monitoring as a second option (see Figure 2). A total of 29.5% would select intermittent monitoring via single‐lead ECG technology, 15.5% 24 h Holter monitoring, and 14.5% would select PPG monitoring. Electrophysiologists were more likely to select intermittent monitoring via a single‐lead ECG technology (42.5% vs. 24%, *p* < 0.001) than non‐electrophysiologists.

### 3.2 Focus on PPG Work‐Up

For each case, respondents who selected PPG as their first diagnostic tool (case 1: 8.9% of respondents; case 2: 9.8% of respondents; case 3: 8.2% of respondents) were presented with three examples of a standard PPG report (60‐s recordings, three measurements per day for 7 days) of either normal measurements, single measurements suggestive of AF, or all measurements suggestive of AF.

**Figure 3:**
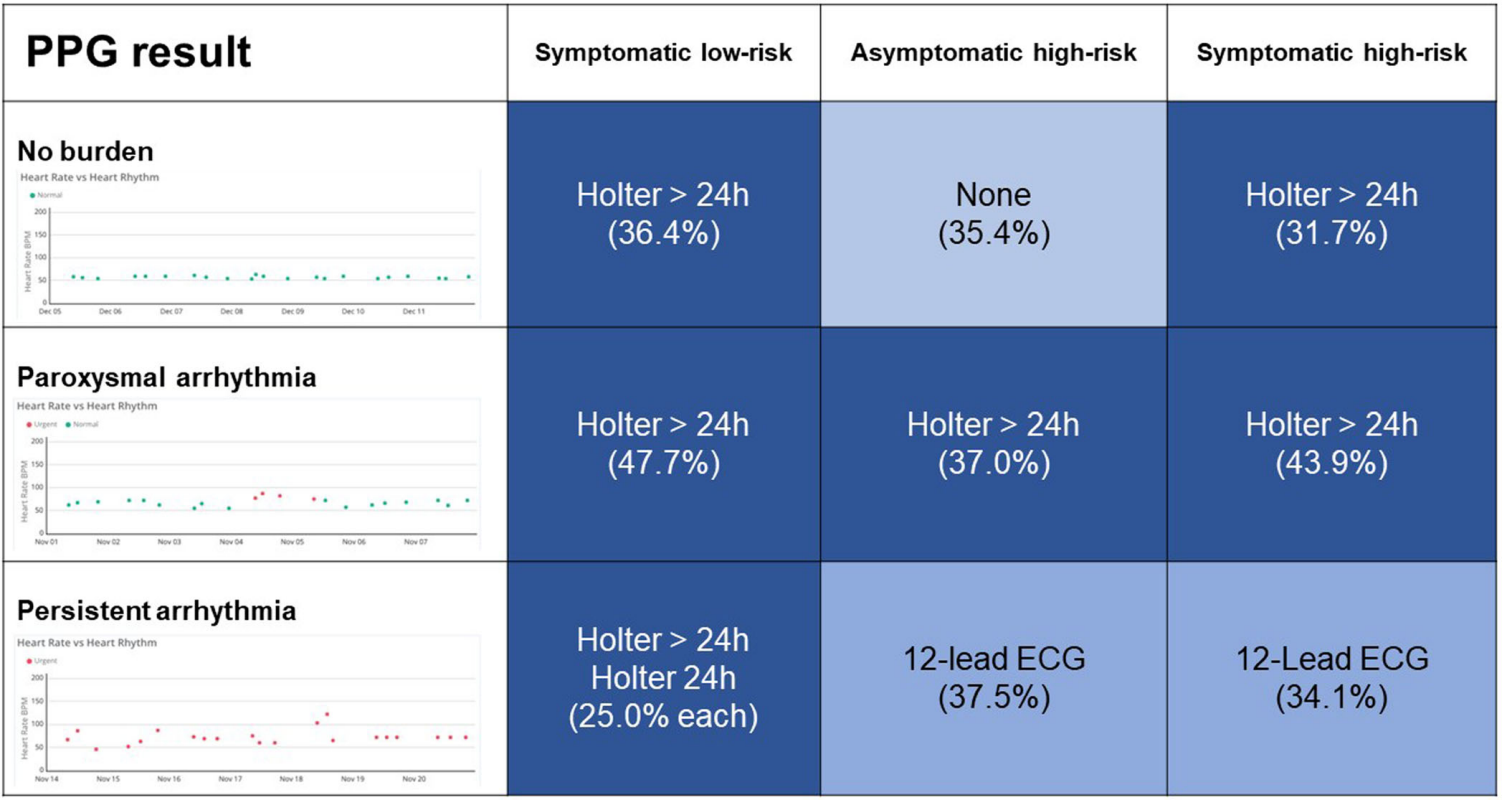
Most common answer for further monitoring in the PPG group, stratified by case and arrhythmia burden. Green dots represent measurements of regular rhythm, and red dots represent measurements suggestive of AF.

The most common answers for further management, stratified by case and arrhythmia burden, are presented in Figure 3.

In a symptomatic low‐risk patient with normal measurements, most respondents (16/44 = 36.4%) would perform > 24 h Holter examination as a next step and 20.5% would perform intermittent single‐lead ECG measurements or not perform further measurements. In case of PPG measurements suggestive of paroxysmal AF, most respondents would try to confirm AF with an ECG‐based measurement (21/44, 47.7% Holter for > 24 h, 20.5% intermittent single‐lead ECG‐based digital device, 18.2% Holter for 24 h, and 4.5% 12‐lead resting ECG). In case of measurements suggestive of persistent AF, most respondents would try to confirm AF with a Holter examination for > 24 h (11/44, 25.0%), with a Holter examination for 24 h (25.0%) or a 12‐lead resting ECG (22.7%).

In an asymptomatic high‐risk patient with PPG measurements not suggestive of AF, most respondents would perform no further workup (17/48 = 35.4%), intermittent monitoring by a single‐lead ECG‐based digital device (25.0%), and a Holter examination for 24 h (16.7%) or > 24 h (14.6%). In case of PPG tracings suggestive of paroxysmal AF, most respondents would try to confirm AF with an ECG‐based measurement (17/46, 37.0% > 24 h Holter, 26.1% intermittent single‐lead ECG, and 21.7% 24 h Holter). In case of measurements suggestive of persistent AF, most respondents would try to confirm AF with a 12‐lead ECG (18/48 = 37.5%), > 24 h Holter examination for 24 h (27.1%), or intermittent single‐lead ECG monitoring (16.7%).

In a symptomatic high‐risk patient with a PPG measurement not suggestive for AF, most respondents would perform > 24 h Holter examination (13/41 = 37.1%) or no other workup (26.8%). Both 12‐lead ECG and intermittent monitoring via single‐lead ECG were recommended at 17.1% each. In case of PPG tracings suggestive of paroxysmal AF, most respondents would try to confirm AF with an ECG‐based measurement (18/41 = 43.9% > 24 h Holter, 29.3% intermittent single‐lead ECG, and 17.1% 24 h Holter). In case of PPG measurements suggestive of persistent AF, most respondents would try to confirm AF with a 12‐lead ECG (14/41 = 34.1%), Holter examination for > 24 h (22.0%) or Holter examination for 24 h (19.5%), or no further monitoring (19.5%).

Both scenarios with PPG tracings suggestive of paroxysmal and persistent AF were presented together with a case vignette (asymptomatic low‐risk patient and symptomatic high‐risk patient) to all respondents. In both cases, respondents would recommend > 24 h Holter recordings (36.4% for tracings suggestive of paroxysmal AF and 47.7% for tracings suggestive of persistent AF). Only 4.5% would confirm AF with a single time point 12‐lead resting ECG after receiving a report of 21/21 PPG tracings suggestive of AF within 1 week.

## 4 Discussion

The DAS‐CAM III participants collected data on physician preferences in using digital devices for the management of AF in over 500 participating physicians within the survey. Our study has four main findings as follows:

– Nearly a third of respondents would not perform AF screening in asymptomatic high‐risk patients.
– Physicians prefer to use ECG‐based (mainly Holter ECGs) over PPG‐based devices to diagnose and screen for AF.
– Physicians use a variety of ECG‐based devices to confirm PPG‐documented tracings suggestive of AF.
– Electrophysiologists would rather use intermittent single‐lead ECG monitoring than traditional Holter ECGs for most clinical indications.

This survey specifically focused on preferred rhythm monitoring strategies in clinical scenarios of patients with different CHA_2_DS_2_‐VASc scores and arrhythmia burdens and symptoms. In symptomatic low‐risk patients, respondents would start with > 24 h Holter recording, in part due to the frequency of symptoms presented in the case. If this failed to diagnose the arrhythmia, most respondents would recommend intermittent single‐lead ECG‐based monitoring. This workup is in line with the EHRA practical guide [1]. However, it requires substantial medical resources compared to the use of wearable devices, which are already available in a substantial proportion of patients in developed countries. ECG documentation of the arrhythmia seems necessary for most individuals in this scenario, and differentiation between specific arrhythmias is crucial in this scenario with a low pretest probability for AF.

In asymptomatic high‐risk patients, most respondents (29.4%) would not perform any monitoring, even though monitoring is indicated by current ESC guidelines for the diagnosis and management of AF [11]. Opportunistic screening for AF in hypertensive patients carries a Class I, Level of Evidence B recommendation in the ESC guidelines, and the EHRA practical guide suggests systematic screening. PPG‐based technology might be a good rule‐out tool in this specific clinical scenario. One explanation for this finding might be the conflicting results from previous large‐scale screening studies including the STROKE‐STOP and the LOOP study [12, 13, 14].

In symptomatic high‐risk patients, most respondents would start by performing a > 24 h Holter recording. This is in line with one of the key messages of the current ESC AF guidelines demanding to “confirm AF” using ECG‐based technologies [11]. It is unclear whether intermittent ECG‐based devices used over longer durations may increase the probability of detecting AF in this patient scenario.

When presented with negative PPG tracings, this was rarely sufficient as a rule‐out for AF, respondents recommended the use of ECG‐based monitoring in these patients. In the case of PPG tracings suggestive of AF, most respondents chose different ECG tools to confirm AF but rarely the cheapest and most effective tool to diagnose AF based on the 1‐week PPG measurement (> 24 h Holter in case of paroxysmal episodes and single 12‐lead ECG in case of persistent episode lasting the whole week). This might also be explained by a lack of experience or trust in the technology.

In 2020, the wEHRAbles 2 survey demonstrated that wearable rhythm device ECG technologies are suitable for AF screening, arrhythmia diagnostics, and patient monitoring [4]. However, only a minority of participants of the wEHRAbles 2 survey found PPG‐based devices suitable for these indications. Since then, new PPG app‐based patient‐centered pathways have been designed, educational content (i.e., PPG dictionary) has been produced, further PPG validation studies have been conducted and screening studies using intermittent or continuous PPG have been conducted [2, 5, 8, 9, 10, 15, 16, 17, 18, 19, 20, 21, 22]. More importantly, the EHRA practical guide “How to use digital devices to detect and manage arrhythmias” currently states that PPG‐based devices can be used for arrhythmia detection in symptomatic patients, in AF screening as well as in integrative AF care pathways [1]. Despite increasing evidence and advancement of the PPG technology and analysis algorithms, this survey with more than 500 respondents demonstrates that just 60% of electrophysiologists and 34% of non‐electrophysiologists have experience with PPG‐based digital devices. Interestingly, older respondents were more often experienced in PPG‐based digital devices than younger respondents. This is also reflected in the small percentage of preferred use of PPG‐based digital devices in the different clinical scenarios assessed in this survey. This survey could not assess the cause for the limited clinical uptake of PPG‐based digital devices, but the lack of large outcome trials demonstrating non‐inferiority of PPG‐based AF diagnosis, clear reimbursement models, and a wider range of educational material such as the TeleCheck‐AF PPG dictionary may partially explain the ongoing skepticism and missing comfort related to the PPG technology [5, 23]. The INTERPRET‐AF survey showed that physicians can detect AF on a PPG output with equivalent accuracy compared to single‐lead ECG if the PPG waveforms are presented together with a tachogram and Poincaré plot, and the quality of the recordings is high [15]. Besides proving and showing the accuracy of PPG‐based technology, also more guidance on how to use PPG‐based digital devices in defined clinical scenarios, such as using a PPG‐based smartphone application on demand around teleconsultation (TeleCheck‐AF approach), may help to increase the implementation of PPG‐based digital devices in the clinic [18].

This survey focused on physician's choices for screening. However, consumer‐driven device development could potentially lead to reversal of the screening process toward first presentation to a physician with a wearable‐detected arrhythmia.

## 5 Limitations

The present survey has limitations attributed to target respondents and questionnaire design. The survey was mainly spread through the network of DAS‐CAM III participants, and participation was voluntary, therefore being prone to selection bias. An online survey might not capture the complexity of screening and monitoring strategies in its whole dimension. To reduce the complexity of the survey, we decided to restrict the patient scenarios to risk profiles and presence of symptoms. In symptomatic patients, we set the symptom burden to twice to avoid favoring one monitoring strategy (i.e., 24 h Holter in a patient with daily symptoms or portable monitor in a patient with monthly symptoms).

## 6 Conclusion

While PPG‐based digital devices are still rarely preferred by physicians in diagnosis and screening for AF, there is a shift toward the use of intermittent monitoring by ECG‐based digital devices, especially in electrophysiologists. More education on the potential of widely available and currently underutilized novel rhythm monitoring tools, such as PPG‐based digital devices, is required to achieve diagnostic pathways as suggested by the EHRA practical guide.

## Ethics Statement

We confirm that all authors have read and approved the manuscript and that no part of the manuscript is published or under consideration elsewhere. The authors confirm that the study was conducted in accordance with the Declaration of Helsinki as revised in 2013.

## Conflicts of Interest

Martin Manninger: Received speaker fees from Bayer, Biosense Webster, Biotronik, Amomed, AOP Orphan, Boston Scientific, Daiichi Sankyo, and BMS/Pfizer and research grants from Biosense Webster and Abbott.

Paweł T. Matusik: Received speech honorarium from Boehringer Ingelheim, Polish Cardiac Society 2018 Scientific Grant in cooperation with Berlin‐Chemie/Menarini (sponsor of the grant: Berlin‐Chemie/Menarini Poland LLC), and participated in educational activities, which were supported by CIED manufacturers as well as Polpharma. None of the declared conflicts are related to the current work.

Vassil Traykov: Declares receiving speaker fees and other honoraria from Boehringer Ingelheim, Astra Zeneca, Berlin Menarini, Abbott, Novartis, Bayer, Merck, Pfizer, and Biotronik.

William F. McIntyre: Received speaking fees from Bayer and Servier and Advisory Fees from Trimedics and AtriCure.

## Supporting information

Supporting information.

## Data Availability

The data that support the findings of this study are available on request from the corresponding author. The data are not publicly available due to privacy or ethical restrictions.
