# Supplementary material for: Physician Preferences in Using Novel Digital Devices for the Management of Atrial Fibrillation—A DAS‐CAM III Survey
Source: Clin Cardiol. 2024 Nov 25;47(12):e24331. doi: 10.1002/clc.24331 (PMC11586575; doi:10.1002/clc.24331)

**Supplemental material**

Supplemental Table 1. Survey questionnaire

| Question | Answer options |
| --- | --- |
| Age (in years) |  |
| Location (country) |  |
| Current profession | - Cardiologist - Electrophysiologist - Cardiology resident - Researcher - Other |
| Have you ever used ECG-based digital technologies for your patients? Or have you ever been consulted or contacted by a patient who used these tools independently? | - Yes - No |
| Have you ever used photoplethysmography (PPG-) based digital technologies for your patients? Or have you ever been consulted or contacted by a patient who used these tools independently? | - Yes - No |
| CASE 1: Which step in routine would you take to detect (or rule out) atrial fibrillation?  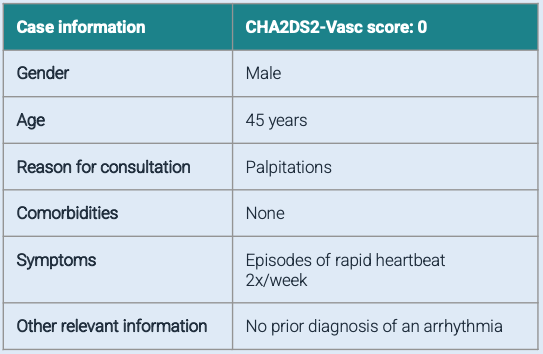 | - Schedule follow-up consultation for an additional 12-lead ECG examination - Schedule follow up consultation for 24h Holter examination - Schedule follow up consultation for >24h Holter examination - Start on-demand, intermittent monitoring via (single-lead) ECG technology - Start on-demand, intermittent monitoring via PPG technology - No further action for now, follow up consult after 1 year - Other |
| CASE 1: SCENARIO 1 if PPG was selected  Which technology would you use to verify the  PPG-based results in this specific patient scenario?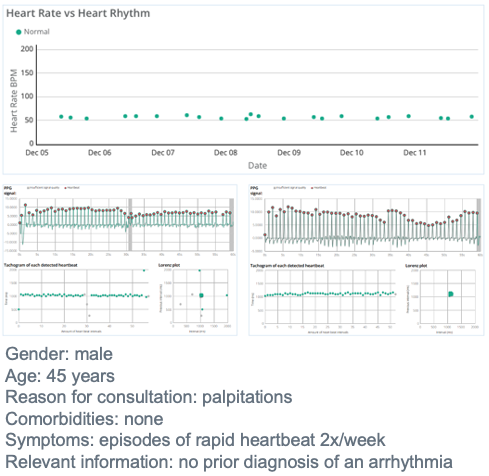 | - 12-lead ECG - 24h Holter examination - >24h Holter examination - Intermittent monitoring via (single-lead) ECG technology - None - Other |
| CASE 1: SCENARIO 2 if PPG was selected  Which technology would you use to confirm the diagnosis of atrial fibrillation in this specific patient scenario?  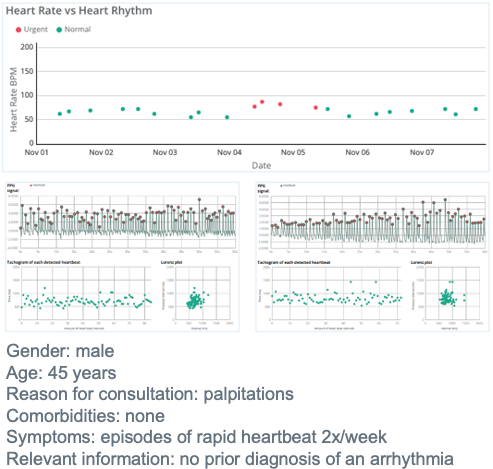 | - 12-lead ECG - 24h Holter examination - >24h Holter examination - Intermittent monitoring via (single-lead) ECG technology - None - Other |
| CASE 1: SCENARIO 3 if PPG was selected  Which technology would you use to confirm the diagnosis of atrial fibrillation in this specific patient scenario?  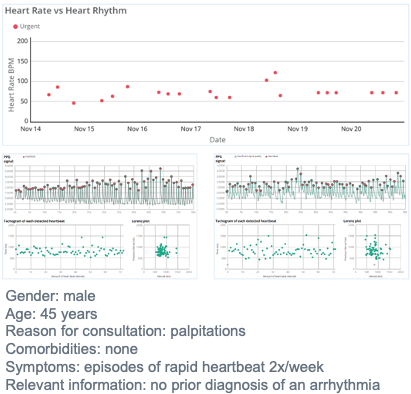 | - 12-lead ECG - 24h Holter examination - >24h Holter examination - Intermittent monitoring via (single-lead) ECG technology - None - Other |
| CASE 1: During the examination, there were no findings suggestive for atrial fibrillation. Which next step in routine would you take to detect (or rule out) atrial fibrillation? | - Schedule follow-up consultation for an additional 12-lead ECG examination - Schedule follow up consultation for 24h Holter examination - Schedule follow up consultation for >24h Holter examination - Start on-demand, intermittent monitoring via (single-lead) ECG technology - Start on-demand, intermittent monitoring via PPG technology - No further action for now, follow up consult after 1 year - Other |
| CASE 2: Which step in routine would you take to detect (or rule out) atrial fibrillation?  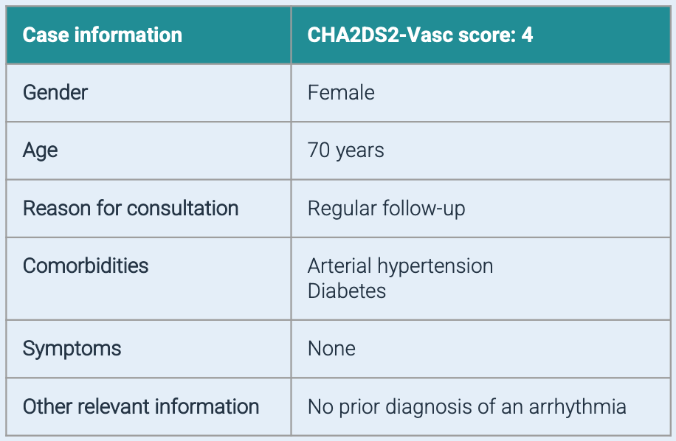 | - Schedule follow-up consultation for an additional 12-lead ECG examination - Schedule follow up consultation for 24h Holter examination - Schedule follow up consultation for >24h Holter examination - Start on-demand, intermittent monitoring via (single-lead) ECG technology - Start on-demand, intermittent monitoring via PPG technology - No further action for now, follow up consult after 1 year - Other |
| CASE 2: SCENARIO 1 if PPG was selected  Which technology would you use to verify the  PPG-based results in this specific patient scenario?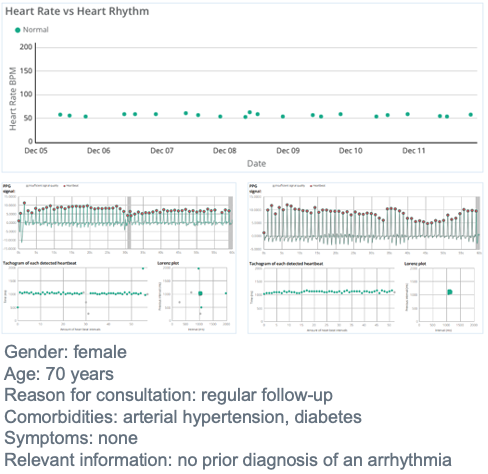 | - 12-lead ECG - 24h Holter examination - >24h Holter examination - Intermittent monitoring via (single-lead) ECG technology - None - Other |
| CASE 2: SCENARIO 2 if PPG was selected  Which technology would you use to confirm the diagnosis of atrial fibrillation in this specific patient scenario?  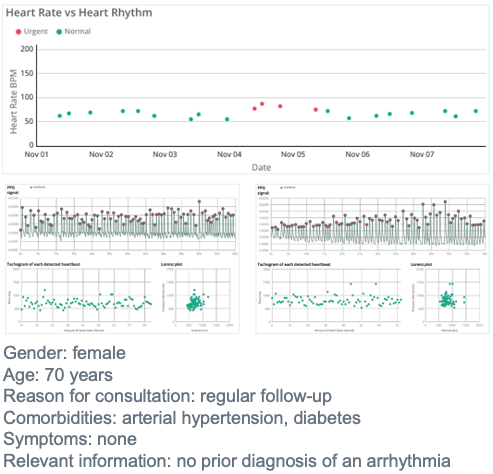 | - 12-lead ECG - 24h Holter examination - >24h Holter examination - Intermittent monitoring via (single-lead) ECG technology - None - Other |
| CASE 2: SCENARIO 3 if PPG was selected  Which technology would you use to confirm the diagnosis of atrial fibrillation in this specific patient scenario?  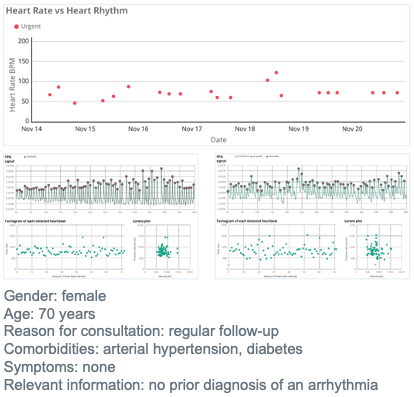 | - 12-lead ECG - 24h Holter examination - >24h Holter examination - Intermittent monitoring via (single-lead) ECG technology - None - Other |
| CASE 2: During the examination, there were no findings suggestive for atrial fibrillation. Which next step in routine would you take to detect (or rule out) atrial fibrillation? | - Schedule follow-up consultation for an additional 12-lead ECG examination - Schedule follow up consultation for 24h Holter examination - Schedule follow up consultation for >24h Holter examination - Start on-demand, intermittent monitoring via (single-lead) ECG technology - Start on-demand, intermittent monitoring via PPG technology - No further action for now, follow up consult after 1 year - Other |
| CASE 3: Which step in routine would you take to detect (or rule out) atrial fibrillation?  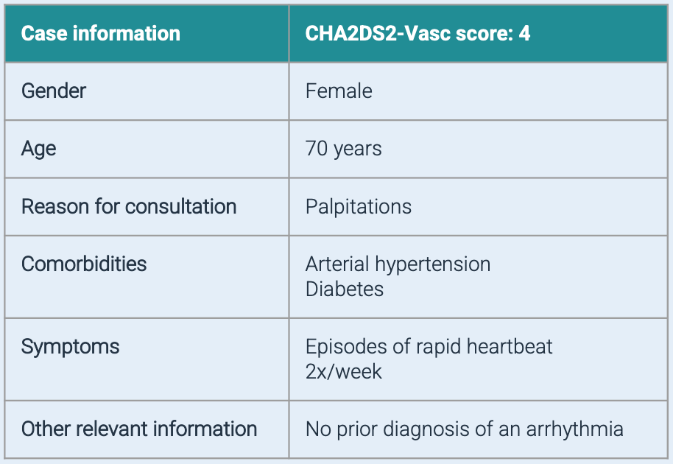 | - Schedule follow-up consultation for an additional 12-lead ECG examination - Schedule follow up consultation for 24h Holter examination - Schedule follow up consultation for >24h Holter examination - Start on-demand, intermittent monitoring via (single-lead) ECG technology - Start on-demand, intermittent monitoring via PPG technology - No further action for now, follow up consult after 1 year - Other |
| CASE 3: SCENARIO 1 if PPG was selected  Which technology would you use to verify the  PPG-based results in this specific patient scenario?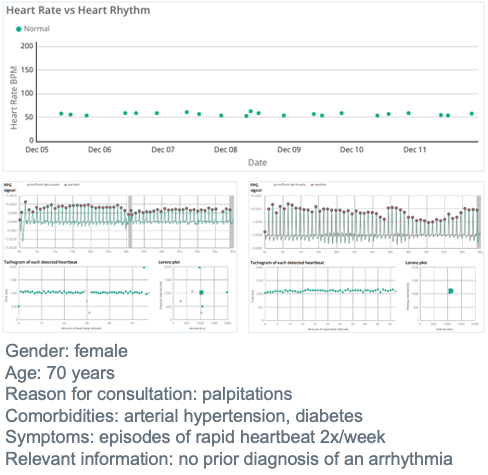 | - 12-lead ECG - 24h Holter examination - >24h Holter examination - Intermittent monitoring via (single-lead) ECG technology - None - Other |
| CASE 3: SCENARIO 2 if PPG was selected  Which technology would you use to confirm the diagnosis of atrial fibrillation in this specific patient scenario?  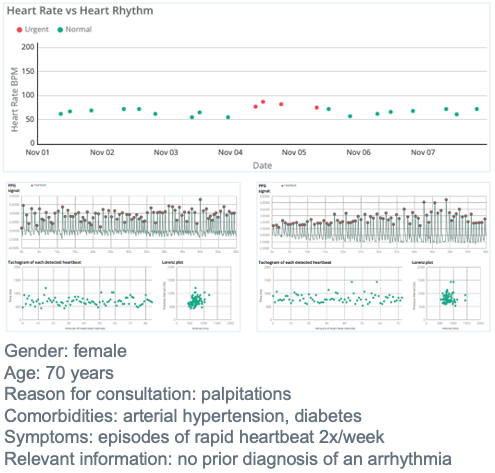 | - 12-lead ECG - 24h Holter examination - >24h Holter examination - Intermittent monitoring via (single-lead) ECG technology - None - Other |
| CASE 3: SCENARIO 3 if PPG was selected  Which technology would you use to confirm the diagnosis of atrial fibrillation in this specific patient scenario?  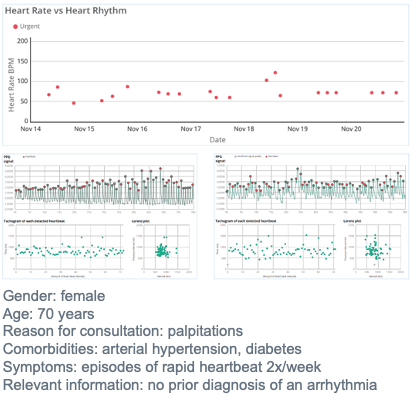 | - 12-lead ECG - 24h Holter examination - >24h Holter examination - Intermittent monitoring via (single-lead) ECG technology - None - Other |
| CASE 3: During the examination, there were no findings suggestive for atrial fibrillation. Which next step in routine would you take to detect (or rule out) atrial fibrillation? | - Schedule follow-up consultation for an additional 12-lead ECG examination - Schedule follow up consultation for 24h Holter examination - Schedule follow up consultation for >24h Holter examination - Start on-demand, intermittent monitoring via (single-lead) ECG technology - Start on-demand, intermittent monitoring via PPG technology - No further action for now, follow up consult after 1 year - Other |
| SCENARIO 1: Which technology would you use to confirm the diagnosis of atrial fibrillation in this specific patient scenario?  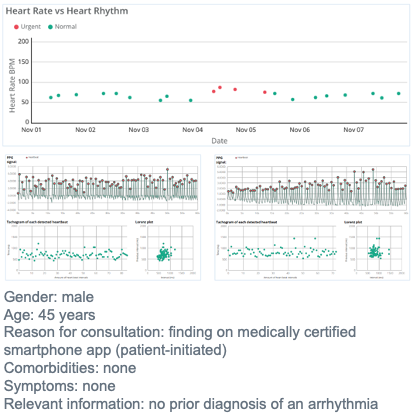 | - 12-lead ECG - 24h Holter examination - Schedule follow up consultation for >24h Holter examination - Intermittent monitoring via (single-lead) ECG technology - None - Other |
| SCENARIO 2: Which technology would you use to confirm the diagnosis of atrial fibrillation in this specific patient scenario?  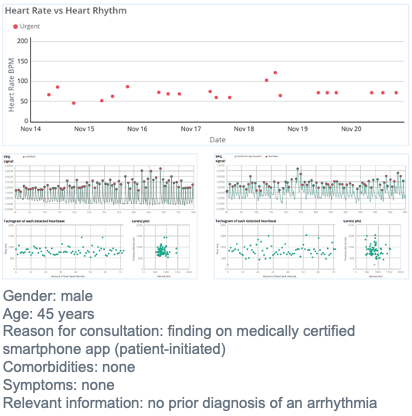 | - 12-lead ECG - 24h Holter examination - Schedule follow up consultation for >24h Holter examination - Intermittent monitoring via (single-lead) ECG technology - None - Other |

Supplemental Figure 1. Consort diagram


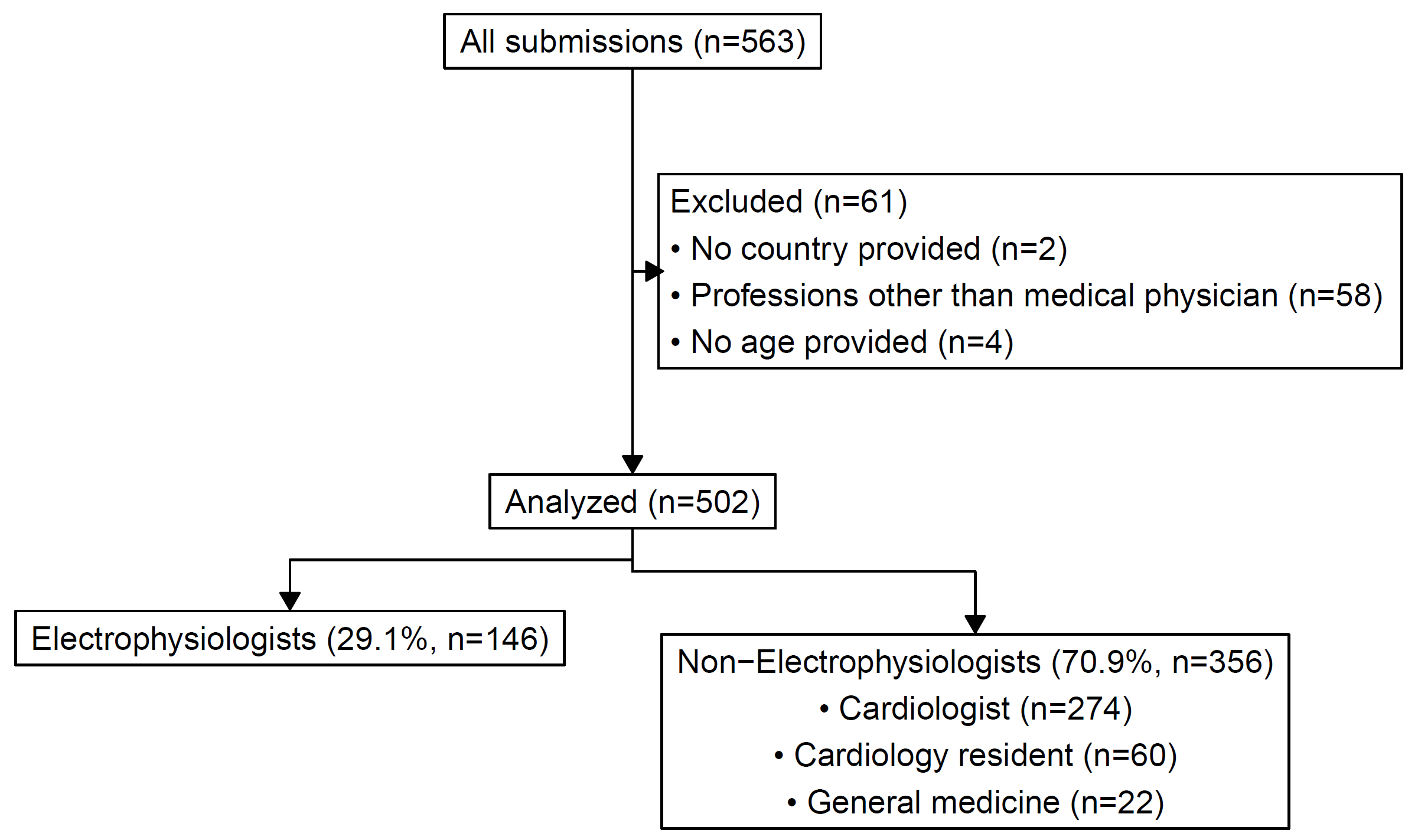

Supplement: Supplementary file 1 — Supporting information. [file CLC-47-e24331-s001.docx]
